# Training allied ophthalmic personnel to meet India's eye care needs

**Published:** 2020-12-31

**Authors:** Sweta Patel, Prem Kumar SG, Pankaj Vishwakarma, Elizabeth Kurian

**Affiliations:** 1 Senior Manager: Programme Impact, Mission for Vision, Mumbai, India.; 2 Manager – Research: Mission for Vision, Mumbai, India.; 3 Head – Programme Impact: Mission for Vision, Mumbai, India.; 4 Chief Executive Officer: Mission for Vision, Mumbai, India.

**A new initiative aims to train young people from disadvantaged backgrounds in India as allied health personnel.**

**Figure:**
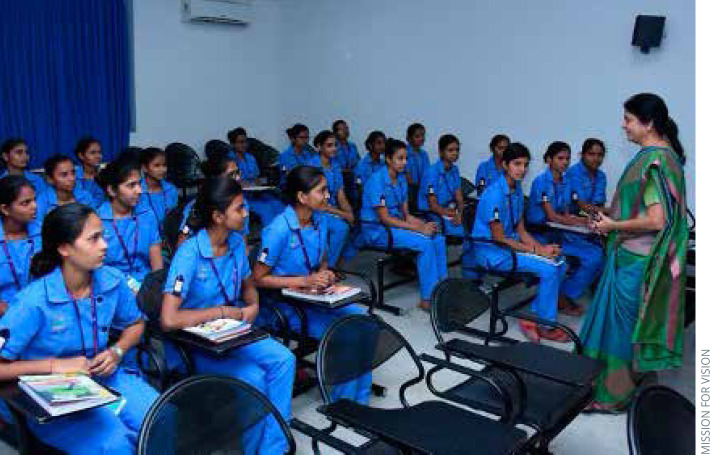
AOP training session for ophthalmic personnel in progress.

There is a need for more skilled and competent allied ophthalmic personnel (AOP) to deliver comprehensive eye health services in India.^1^,^2^

Mission Saksham is an initiative that aims to improve eye care delivery and reduce poverty by offering free AOP training to young men and women from economically vulnerable communities.

## Scholarship program

Mission for Vision in partnership with the Wen Giving Foundation aims to train more allied ophthalmic personnel in India by offering scholarships to people from economically vulnerable and marginalised communities. The first phase aspires to train 2,000 candidates by 2025.

The broad objectives of Mission Saksham^3^ are to:

1. Strengthen the capacity of allied ophthalmic personnel in the country:
  - As of January 2021, a total of 236 (female: 190 and male: 46) students were enrolled in various ophthalmic courses at four tertiary eye care institutions in India. Periodic monitoring of the ongoing course by project staff, parameters like the attendance of the students and grades scored are used a proxy indicators of quality. Phase - II of this initiative involves collaborating with three new partner eye care institutions, to train about an additional 250 AOPs.
2. Standardising the teaching curriculum and training programs:
  - In the absence of a central/national accreditation body in India, it is hoped that every hospital will adopt its curriculum, examination patterns and teaching modalities. Under Mission Saksham, we looked at 35 different AOP courses across the country and found that about 80% do not have accreditation. Efforts are underway to encourage these hospitals to obtain local accreditation for the courses.
  - Mission Saksham invests in building the capacities of partner eye care institutions in the country, including standardising of their AOP training programmes. Consequently, Mission Saksham identified a total of 14 partner eye care institutions, and efforts are underway to build their capacities by conducting regular workshops. Efforts are ongoing, in partnership with several leading eye care centres in the country, to build institutional capacity through the development of standardised curriculum, course material, course design, and faculty training.
3. Empowering youth from challenging backgrounds - especially those from smaller towns and rural background.
  - The potential AOP candidates to be trained are selected from economically weaker backgrounds. The identification and selection of candidates is made in partnership with the local partner eye hospital; candidates whose monthly family/household income is less than or equal to Rs. 10,000 (roughly US $135), are given priority. About 70% of place is reserved for women candidates. Scholarships are provided to the selected candidates, to cover tuition fees, monthly stipend, free boarding and food facilities, and supplies including uniforms, books and stationery.

## Initial impact and way forward

The first group of 11 AOPs completed the training in 2019 and was placed in a partner hospital. Hailing from modest backgrounds, these trained AOPs now earn between Rs. 7,000 and 10,000 (the US $96–137) every month, contributing additional income to their households. Also the 29 students who have graduated in the 2019-2020, have served a total of 24,600 patients in those years combined.

In future, mission for vision will identify, partner, and network with new eye care institutions, to train AOP candidates in the country.
